# IL-17A expression by both T cells and non-T cells contribute to HSV-IL-2-induced CNS demyelination

**DOI:** 10.3389/fimmu.2023.1102486

**Published:** 2023-02-02

**Authors:** Satoshi Hirose, Shaohui Wang, Ujjaldeep Jaggi, Harry H. Matundan, Mihoko Kato, Xue-Ying Song, Sara J. Molesworth-Kenyon, Robert N. Lausch, Homayon Ghiasi

**Affiliations:** ^1^ Center for Neurobiology & Vaccine Development, Ophthalmology Research, Department of Surgery, Cedars-Sinai Medical Center, Los Angeles, CA, United States; ^2^ Department of Biology, Pomona College, Claremont, CA, United States; ^3^ Applied Genomics, Computation, and Translational Core, Cedars-Sinai Medical Center, Los Angeles, CA, United States; ^4^ Department of Natural Sciences, University of West Georgia, Carrollton, GA, United States; ^5^ Department of Microbiology and Immunology, University of South Alabama, College of Medicine, Mobile, Al, United States

**Keywords:** Ocular infection, mouse, HSV-IL-2, knockout, IL17RA, IL17RC, IL17RD

## Abstract

Previously we reported that a recombinant HSV-1 expressing murine IL-2 (HSV-IL-2) causes CNS demyelination in different strains of mice and in a T cell-dependent manner. Since T_H_17 cells have been implicated in CNS pathology, in the present study, we looked into the effects of IL-17A^-/-^ and three of its receptors on HSV-IL-2-induced CNS demyelination. IL-17A^-/-^ mice did not develop CNS demyelination, while IL-17RA^-/-^, IL-17RC^-/-^, IL-17RD^-/-^ and IL-17RA^-/-^RC^-/-^ mice developed CNS demyelination. Adoptive transfer of T cells from wild-type (WT) mice to IL-17A^-/-^ mice or T cells from IL-17A^-/-^ mice to Rag^-/-^ mice induced CNS demyelination in infected mice. Adoptive T cell experiments suggest that both T cells and non-T cells expressing IL-17A contribute to HSV-IL-2-induced CNS demyelination with no difference in the severity of demyelination between the two groups of IL-17A producing cells. IL-6, IL-10, or TGFβ did not contribute to CNS demyelination in infected mice. Transcriptome analysis between IL-17A^-/-^ brain and spinal cord of infected mice with and without T cell transfer from WT mice revealed that “neuron projection extension involved in neuron projection guidance” and “ensheathment of neurons” pathways were associated with CNS demyelination. Collectively, the results indicate the importance of IL-17A in CNS demyelination and the possible involvement of more than three of IL-17 receptors in CNS demyelination.

## Introduction

Optic neuritis involves primary inflammation of the optic nerve and is associated with loss of vision or double vision, and painful ocular movements (1–3). Several lines of evidence indicate that it is a multifactorial, autoimmune condition involving T-cell mediated demyelination of the optic nerve. In common with other such diseases (e.g., MS, psoriasis, inflammatory bowel disease, rheumatoid arthritis), it has proven difficult to distinguish causative responses from bystander effects. Epidemiologic studies have implicated environmental factors as a contributing factor, including infectious agents such as certain viruses (e.g., HCMV, EBV, HHV-6, HHV-7, JCV, HTLV-1, SFV, TMEV) (4, 5). However, this concept remains controversial (6–8) and, if an infectious agent is involved, it alone may not be sufficient to initiate the observed pathology. Optic neuritis is of particular interest due to its association with multiple sclerosis (MS). The visual pathway is one of the early and most commonly affected sites in MS patients in which optic nerves are demyelinated causing blurred or “washed-out vision” (9–13). In about 50-70% of patients with monosymptomatic optic neuritis, clinically silent MS-like lesions are found on brain MRI (2), and an optic neuritis episode is a strong predictor for development of MS (14, 15). More recent prospective studies have demonstrated that, with extended follow-up, at least half of the patients with monosymptomatic optic neuritis will be diagnosed with MS (2, 3, 16–18). This close relationship between optic neuritis and MS has made the optic nerve an MS-relevant issue, and the autoimmune responses in optic neuritis may reflect early events that occur in human MS.

To provide a framework for analysis of potential multifactorial interactions, we developed a mouse model of optic neuritis that combines overexpression of interleukin-2 (IL-2) in the context of HSV-1 infection using the recombinant HSV-IL-2 virus (19). Neither ocular HSV-1 infection alone nor overexpression of IL-2 alone induces optic nerve or CNS pathology. However, ocular infection with this recombinant HSV-1 that constitutively overexpresses cytokines demonstrated that HSV-IL-2 (but not HSV-IL-4, HSV-IFN-γ, HSV-IL-12p35 or HSV-IL-12p40) elicited optic neuropathy as determined by changes in visual-evoked cortical potentials (VECPs) and typical pathologic changes in the optic nerve and CNS (20–23). In our previous studies, knockout, depletion, and adoptive transfer approaches indicated that both CD4^+^ and CD8^+^ T cells contribute to the HSV-IL-2-induced optic nerve and CNS pathology (20, 24). Characterization of HSV-IL-2-infected cells revealed the dysregulation of cytokines and the inhibition of T_H_1 differentiation which had the potential to lead to production of pathogenic T cells (24, 25). It is well established that both IL-17 producing CD4^+^ (26) and CD8^+^ T cells (27) play important roles in autoimmunity. T_H_17 cells have been implicated in auto-aggressive CNS pathology, and studies suggest that these cells orchestrate inflammation in diseases including psoriasis, inflammatory bowel disease, and rheumatoid arthritis (RA). T_H_17 cells trigger nerve damage in experimental autoimmune encephalomyelitis (EAE), and brain lesions of multiple sclerosis (MS) patients exhibit high levels of IL-17. IL-2 has been shown to promote T_H_17 development *in vivo* (28). In addition to T cells (26, 27), innate lymphoid cells (ILCs), CD4^−^CD8^−^ T cells, γδT cells, invariant natural killer (iNK) T cells, NK cells, neutrophils, mast cells and B cells have been shown to secrete IL-17 (29–34).

In this study we investigated the contribution of IL-17A and three of its receptors to the development of CNS demyelination within an HSV-IL-2 model of ocular neuritis. Our data suggests that: **(1)** IL-17A is involved in HSV-IL-2-induced CNS demyelination; **(2)** production of IL-17A by both T cells and non-T cells is contributing to CNS pathology; **(3)** absence of either the IL-17RA, IL-17RC, IL-17RARC, or IL-17RD receptor does not block CNS demyelination in HSV-IL-2 infected mice; **(4)** IL-6, IL-10, or TGFβ do not contribute to CNS demyelination in HSV-IL-2 infected mice; and **(5)** down regulation of genes associated with neuron projection, extension, and guidance may have contributed to demyelination, while regulation of genes associated with the pathway for ensheathment of neurons may contributed to remyelination. Collectively, these data suggest that elevation of IL-2 levels in the context of viral infection drives an auto-aggressive IL-17A response that causes optic neuritis and CNS pathology. Both T cells and non-T cells contribute to the production of IL-17A, which then affects T-cell lineage commitment and shifts the overall response to that of an auto-aggressive T_H_17 response.

## Materials and methods

### Ethics statement

All animal procedures were performed in strict accordance with the Association for Research in Vision and Ophthalmology Statement for the Use of Animals in Ophthalmic and Vision Research and the NIH *Guide for the Care and Use of Laboratory Animals* (ISBN 0-309-05377-3). The animal research protocols were approved by the Institutional Animal Care and Use Committee of Cedars-Sinai Medical Center (Protocols # 6134 and 9833).

### Cells and virus

RS (rabbit skin) cells were generated in our laboratory, prepared, grown in MEM media plus 5% FBS and used as we described previously (35, 36). Triple plaque-purified HSV-IL-2 and McKrae (parental virus for HSV-IL-2) were grown in RS cell monolayers as described previously (19, 37).

### Mice

Inbred female C57BL/6J, Rag1^-/-^, Rag2^-/-^, IL-6^-/-^ and IL-10^-/-^ mice were obtained from the Jackson Laboratory (Bar Harbor, ME). IL-17A^-/-^, CD11cdnTGF-RII, and CD4dnTGF-RII mice were described previously (38–40). IL-17RC^-/-^ mice were developed by Genentech and were obtained from the Mutant Mouse Regional Resource Center (University of California, Davis). IL-17RA^-/-^ and IL-17RA^-/-^RC^-/-^ were developed in our Lab and described previously (41). IL-17RD^-/-^ mice were obtained from the Mutant Mouse Regional Resource Center (University of California, Davis). All mice used in this study have a B6 background and were bred in-house. Only female (6 to 8-wk-old) mice were used in the study as male mice are more resistance to HSV-IL-2-induced CNS demyelination than female mice (21). This is comparable to observations made in other models for MS.

### Ocular infection

IL-17A^-/-^, IL1-7RA^-/-^, IL-17RC^-/-^, IL-17RA^-/-^RC^-/-^, IL-17RD^-/-^, Rag1^-/-^, Rag2^-/-^, IL-6^-/-^, IL-10^-/-^, CD11cdnTGF-RII, CD4dnTGF-RII and WT control mice were infected ocularly with 2 X 10^5^ PFU of HSV-IL-2 or WT HSV-1 strain McKrae as a control in 2μl of tissue culture media as an eye drop without corneal scarification as we have described previously (42).

### Preparation of brain, spinal cord, and optic nerves for histological analyses

In our previous studies we looked at CNS demyelination in brain, spinal cord and optic nerves of HSV-IL-2 infected mice on days 5, 7, 14, 18, and 75 post infection (PI) and detected demyelination as early as day 14 PI. Thus, in this study brain, spinal cord, and optic nerves of infected mice were removed at necropsy on day 14 PI. Isolated tissues were snap frozen in an isopentane-liquid nitrogen bath and stored at –80**^°^** C. Transverse sections 8-10 μm thick of the entire brain, spinal cord, or optic nerve were cut, air-dried overnight, and fixed in acetone for 3 min at 25**^°^** C (21, 24, 43). Consecutive sections were used for the pathological analyses.

### Analysis of demyelination using Luxol Fast Blue staining

The presence or absence of demyelination in infected mice was evaluated using LFB staining of formalin-fixed sections of optic nerve as we described previously (20). Every fifth section of brain, spinal cord and optic nerve was stained for LFB. The number of plaques, size of plaques, and shape of plaques on multiple fields were evaluated by investigators who were blinded to the treatment groups using serial sections of CNS tissues. The amount of myelin loss in the stained sections of brain, spinal cord and optic nerve was measured using the NIH Image J software analysis system. The areas of demyelination (clear-white) to normal tissue (blue) were quantified using 150 random sections from the brain and spinal cord, or 30 sections from the optic nerve of each animal. Demyelination in each section was confirmed by monitoring adjacent sections. The percentage of myelin loss was calculated by dividing the lesion size into the total area for each section.

### Adoptive transfer of T cells

Total T cells from WT C57BL/6 or IL-17A^-/-^ donor mice were isolated using magnetic beads as described by the manufacturer (Miltenyi Biotec, Auburn, CA). Each recipient Rag1^-/-^, Rag2^-/-^ or IL-17A^-/-^ mouse was intraperitoneally injected once with T cells equivalent to one donor mouse in 300 μl of MEM. The recipient mice were infected ocularly with HSV-IL-2 or control virus 2 weeks after the transfer of the cells.

### Library preparation and sequencing

Brain and spinal cord from HSV-IL-2 infected IL-17A^-/-^ and IL-17A^-/-^ mice that received WT T cells were isolated on day 14 post ocular infection. Total RNA from brain and spinal cord of infected mice were isolated using a SMART-Seq V4 Ultra Low RNA Input Kit for Sequencing (Takara Bio USA, Inc., Mountain View, CA). Isolated RNA was used to generate double-stranded cDNA by reverse transcription for library preparation using the Nextera XT Library Preparation kit (Illumina, San Diego, CA). cDNA was quantitated using Qubit (Thermo Fisher Scientific). cDNA normalized to 80 pg/µl was fragmented and sequencing primers were added simultaneously. A limiting-cycle PCR added Index 1 (i7) adapters, Index 2 (i5) adapters, and sequences required for cluster formation on the sequencing flow cell. Indexed libraries were pooled, washed, and the pooled library size was verified using a 2100 Bioanalyzer (Agilent Technologies, Santa Clara, CA) and quantified using Qubit. Libraries were sequenced using a NextSeq 500 (Illumina) with a 1x75 bp read length and coverage of over 25M reads/sample.

### Data analysis

Raw sequencing data was demultiplexed and converted to fastq format by using bcl2fastq v2.20 (Illumina, San Diego, California) and reads were aligned to the transcriptome using STAR (version 2.6.1) (44) and RSEM (version 1.2.28) (45) with default parameters, using a custom mouse GRCm38 transcriptome reference downloaded from http://www.gencodegenes.org, containing all protein coding based on mouse GENCODE version 24 annotation and HSV-1 gene (MN136524). Expression counts for each gene in all samples were normalized by a modified trimmed mean of the M-values normalization method. Each gene was fitted into a negative binomial generalized linear model, and the Wald test was applied to assess the differential expressions between two sample groups by DESeq2 (version 1.26.0) (46). Benjamini and Hochberg procedure was applied to adjust for multiple hypothesis testing, and differential expression gene (DEG) candidates were selected with a false discovery rate less than 0.1 or 0.05. DEG candidates were used for GO/KEGG enrichment analysis performed with ClusterProfileR (47).

### Statistical analyses

Student’s t test and ANOVA were performed using the computer program Prism (GraphPad, San Diego, CA). Results were considered statistically significant when the “P” value was <0.05.

## Results

### Absence of demyelination in the CNS of IL-17A^-/-^ mice infected with HSV-IL-2

**Figure 1:**
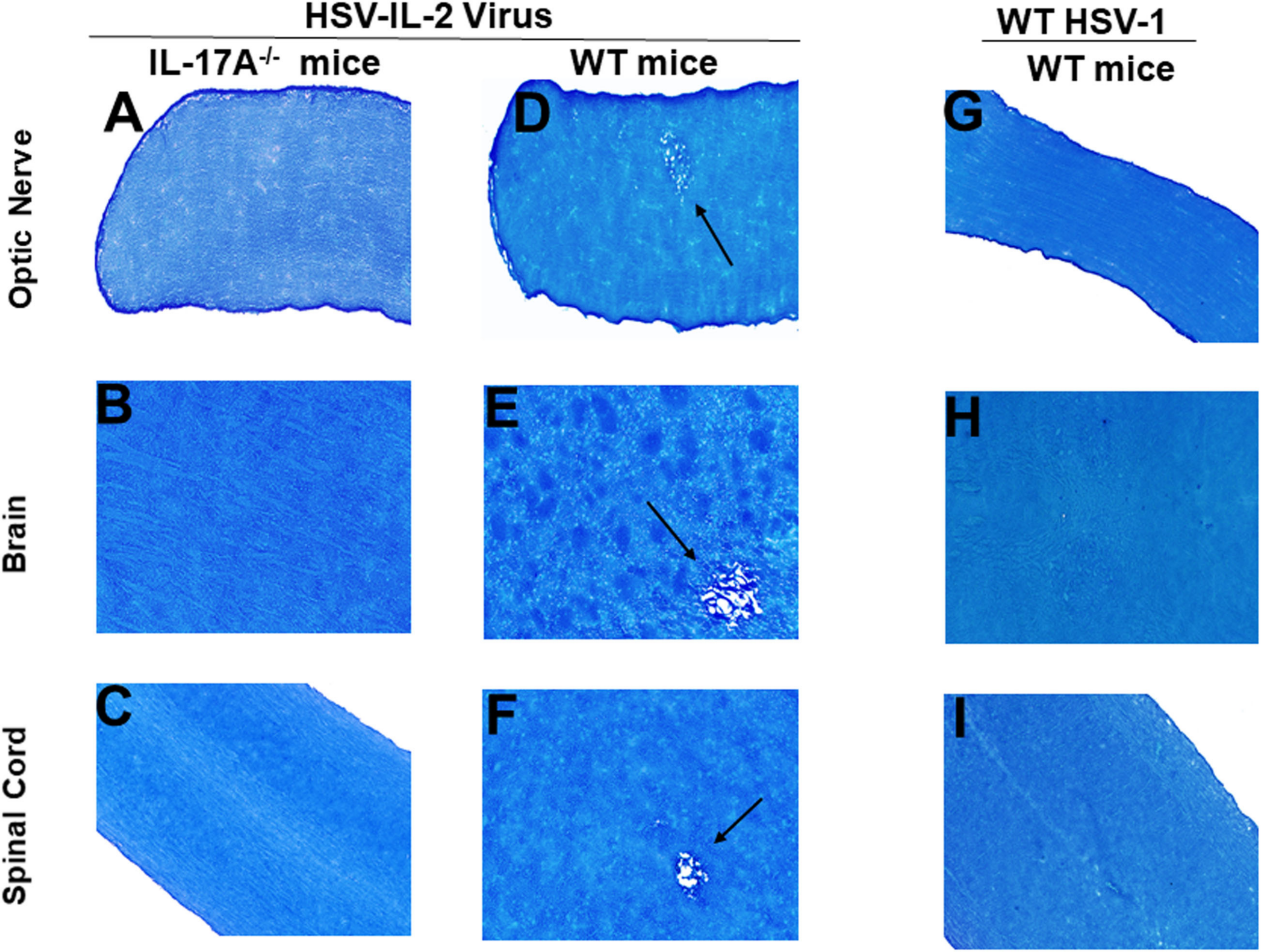
Role of IL-17A in HSV-IL-2 induced CNS demyelination. Five female IL-17A^-/-^ **(A–C)** and WT C57BL/6 mice were infected ocularly with 2 X10^5^ pfu/eye of HSV-IL-2 or WT virus as described in Materials and Methods. On day 14 PI, optic **(A, D, G)** nerves, brain **(B, E, H)**, and spinal cord **(C, F, I)** were collected, fixed, sectioned, and stained with LFB. Representative photomicrographs are shown. Arrows indicate areas of demyelination. x10 objective lens was used (100x mag.).

Previously we have shown that different strains of mice, independent of their MHC background, are susceptible to HSV-IL-2-induced CNS demyelination, but not to WT virus or recombinant HSV-1 expressing murine IL-4, IL-12p35, IL-12p40 or IFN-γ (20, 21, 24, 43). T_H_17 cell involvement has also been described in many autoimmune diseases (48–56). Thus, to determine what role T_H_17 plays in our model of CNS demyelination, five 6-8 week old female IL-17A^-/-^ mice of a C57BL/6 background were infected with 2 x 10^5^ PFU/eye of HSV-IL-2 or WT McKrae control virus. Age matched female C57BL/6 mice were used as an experimental control. On day 14 post-infection (PI), optic nerve, brain and spinal cord samples were dissected. Samples were sectioned, fixed and stained with Luxol fast blue (LFB) as we described previously (20, 21, 24, 43). Representative photomicrographs of optic nerve, brain, and spinal cord sections are shown in **Figure 1**. No demyelination was observed in optic nerves (**Figure 1A**), brain (**Figure 1B**) or spinal cord (**Figure 1C**) of IL-17A^-/-^ mice infected with HSV-IL-2 virus. As expected, demyelination was observed in the optic nerves (**Figure 1D**), brain (**Figure 1E**) or spinal cord (**Figure 1F**) of WT mice infected with HSV-IL-2 virus. No such lesions or other signs of demyelination were observed at this time point in optic nerves (**Figure 1G**), brain (**Figure 1H**) or spinal cord (**Figure 1I**) of the WT mice infected with HSV-1 strain McKrae. These results suggest that the absence of IL-17A blocks HSV-IL-2-induced CNS demyelination in infected mice and confirms the requirement for the overexpression of IL-2 for CNS demyelination within the model.

### Role of IL-17A expressing T cells and non-T cells during CNS demyelination

**Figure 2:**
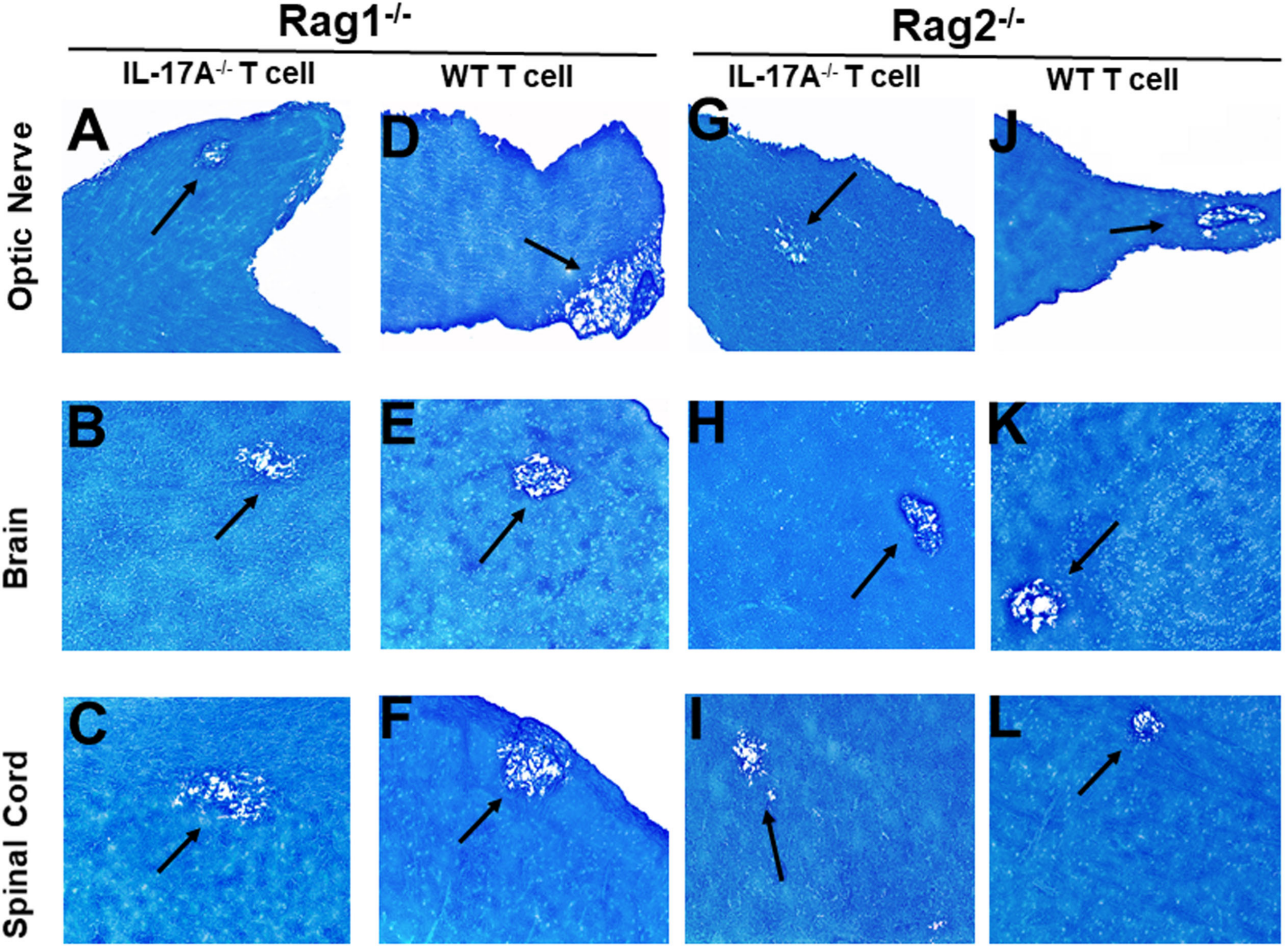
Adoptive transfer of IL-17A^-/-^ and WT T cells to Rag mice. Naive T cells were isolated from five IL-17A^-/-^ and WT mice using a magnetic bead system. Isolated T cells were adoptively transferred *via* intraperitoneal injection (i.p.) into five Rag1^-/-^ and Rag2^-/-^ mice. Two weeks post-adoptive transfer, mice were infected ocularly with 2 X10^5^ pfu/eye of HSV-IL-2 virus. On day 14 PI, optic nerves **(A, D, G, J)**, brain **(B, E, H, K)**, and spinal cord **(C, F, I, L)** samples were harvested, sectioned, and stained with LFB. The left panel depicts a Rag1^-/-^ mouse transferred with IL17A^-/-^ T cells **(A, B, C)** or wild type T cells **(D, E, F)**, respectively. The right panel depicts a Rag2^-/-^ mouse transferred with IL17A^-/-^ T cells **(G, H, I)** or wild type T cells **(J, K, L)**, respectively. Representative sections are shown with arrows indicating areas of demyelination. x10 objective lens was used (100x mag.).

**Figure 3:**
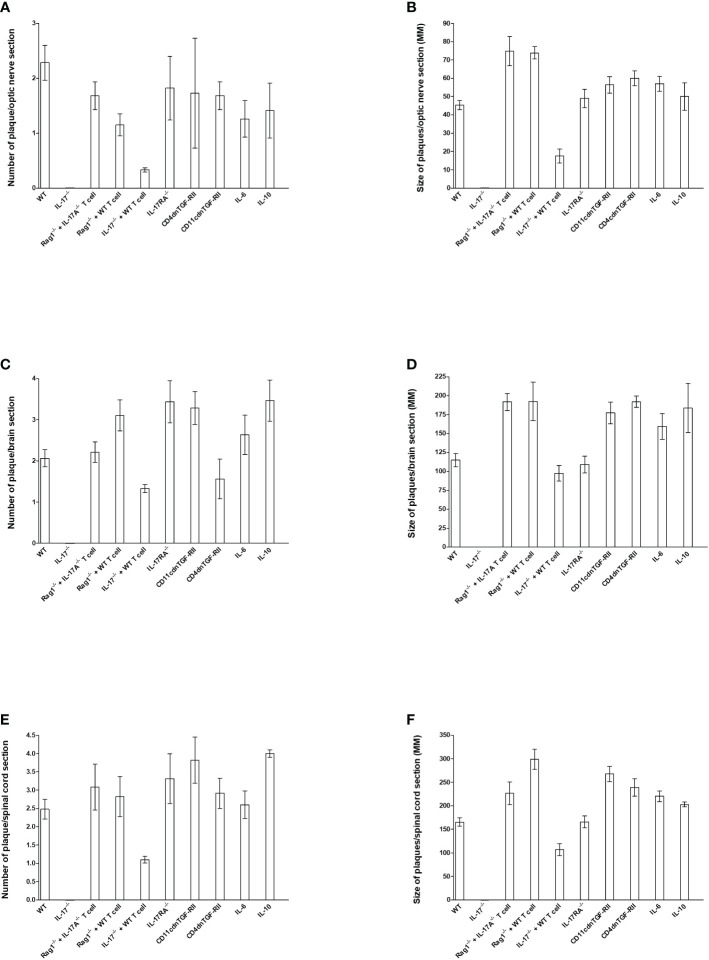
Number and size of demyelination plaques in CNS of infected HSV-IL-2 mice. The entire brain, spinal cord, and optic nerves of each of the 5 animals described in **Figures 1**, **2**, **4**–**6** were sectioned and every 5th slide of each tissue was stained. The number **(A, C, E)** and size of **(B, D, F)** demyelination plaques in the entire sections of brain, spinal cord **(E, F)** and optic nerves **(A, B)** were counted and measured, respectively. Data are presented as number of plaques per total sections or size of plaques per total sections. Data are presented as mean number of demyelination plaques or mean of size of demyelination plaques using a total of 150 sections for brain and spinal cord and 30 sections for optic nerve from 5 mice per group.

Both CD4^+^ and CD8^+^ T cells produce IL-17A and play important roles in autoimmunity (26, 27). Previously we have shown that both CD4^+^ and CD8^+^ T cells are involved in CNS demyelination (24). To test the hypothesis that IL-17A-producing T cells are contributing to CNS demyelination, T cells were isolated using magnetic beads from naive female IL-17A^-/-^ mice or WT C57BL/6 mice. The cells were then adoptively transferred into Rag1^-/-^ or Rag2^-/-^ mice *via* intraperitoneal injection (i.p.). Rag1^-/-^ or Rag2^-/-^mice are characterized by a complete block in T and B cell development. Two weeks after adoptive transfer, all the Rag1^-/-^ and Rag2^-/-^ recipient mice were infected ocularly with HSV-IL-2 virus as above. Fourteen days after infection (day 14 PI), the mice were sacrificed and optic nerves, brain and spinal cord were removed, sectioned, fixed and stained with LFB. Representative photomicrographs are shown in **Figure 2**. A summary of the data concerning the numbers and size of demyelination plaques within the optic nerve, brain and spinal cord sections from these mice are shown in **Figure 3**.

Rag1^-/-^ mice that received T cells from IL-17A^-/-^ mice showed demyelination in their optic nerve (**Figure 2A**), brain (**Figure 2B**) and spinal cord (**Figure 2C**). Similarly, Rag1^-/-^ mice that received T cells from WT mice also showed demyelination in their optic nerve (**Figure 2D**), brain (**Figure 2E**) and spinal cord (**Figure 2F**). Similar to Rag1^-/-^ mice, Rag2^-/-^ mice that received T cells from IL-17A^-/-^ mice showed demyelination in their optic nerve (**Figure 2G**), brain (**Figure 2H**) and spinal cord (**Figure 2I**). As expected, demyelination also was detected in optic nerve (**Figure 2J**), brain (**Figure 2K**) and spinal cord (**Figure 2L**) of Rag2^-/-^ mice that received T cells from WT mice. These results suggest that the absence of IL-17A expression in T cells is not adequate to prevent CNS demyelination.

**Figure 4:**
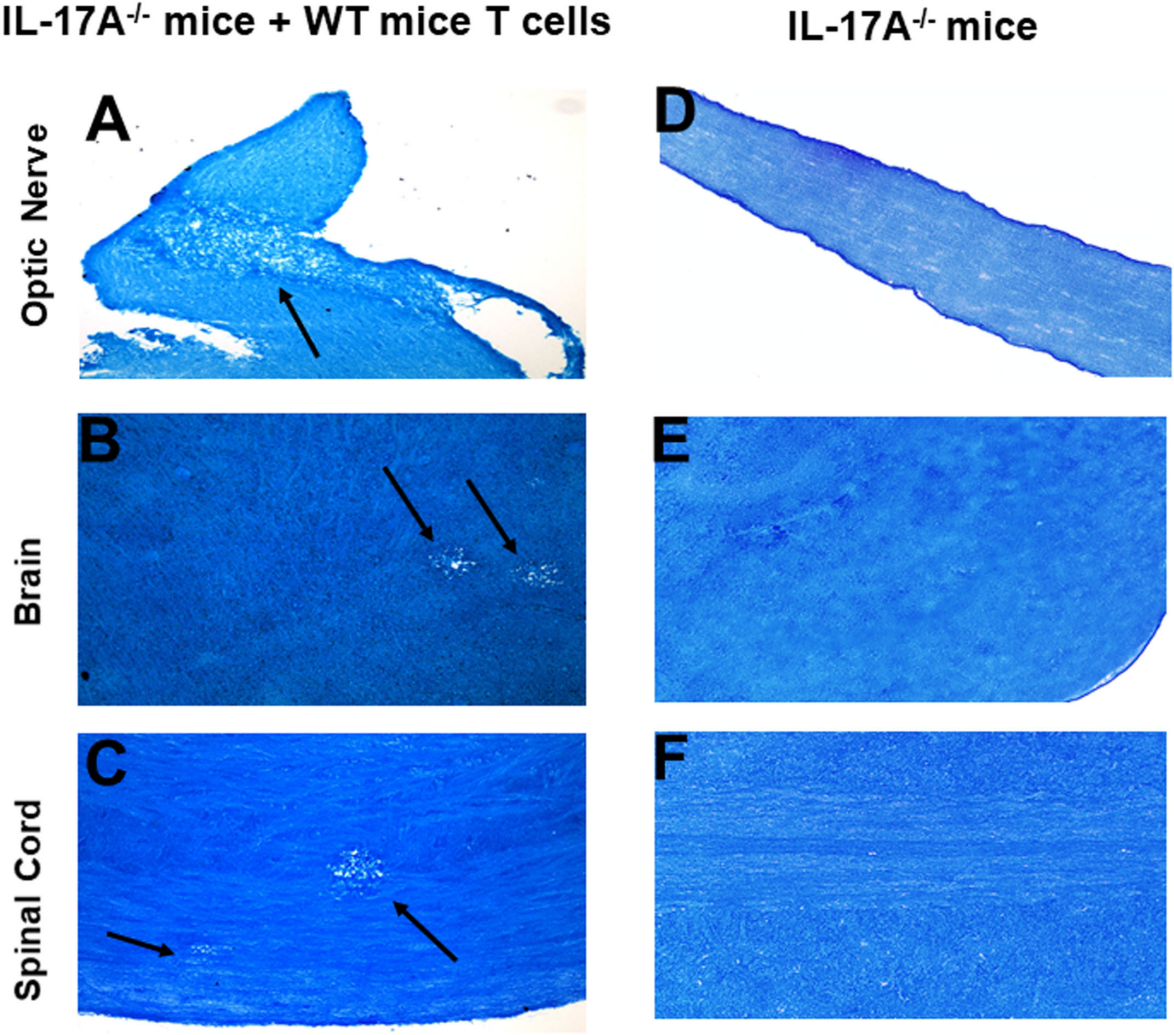
Adoptive transfer of WT T cells to IL-17A^-/-^ mice. Naive T cells were isolated from 5 WT mice. Magnetically isolated cells were transferred by intraperitoneal injection into 5 recipient IL-17A^-/-^ mice. Two weeks post-adoptive transfer, recipient mice **(A, B, C)** and control IL-17A^-/-^ mice without T cells transfer **(D, E, F)** were infected ocularly with 2 X10^5^ pfu/eye of HSV-IL-2 virus. Representative optic nerves **(A, D)**, brain **(B, E)**, and spinal cord **(C, F)** on day 14 PI from infected mice are shown. Arrows indicate areas of demyelination. x10 objective lens was used (100x mag.).

In addition to T cells, other cell types such as ILCs, CD4^−^CD8^−^ T cells, γδT cells, invariant natural killer (iNK) T cells, NK cells, neutrophils, mast cells and B cells express IL-17A (29–34). Thus, we next tested if transfer of T cells from WT mice to IL-17A^-/-^ mice could induce CNS demyelination after HSV-IL-2 infection. T cells were isolated using magnetic beads from naive female WT C57BL/6 mice and the cells injected intraperitoneally into IL-17A^-/-^ recipient mice. Two-weeks after adoptive transfer, all the recipient mice and control IL-17A^-/-^ mice without T cells transfer were infected ocularly with HSV-IL-2 virus. Fourteen days post infection, the mice were sacrificed, and optic nerves, brain and spinal cord were removed, sectioned, fixed and stained with LFB. Representative photomicrographs are shown in **Figure 4**, and a summary of the data concerning the numbers and size of demyelination of the optic nerve, brain and spinal cord sections from mice infected with HSV-IL-2 are shown in **Figure 3**. IL-17A^-/-^ mice that received T cells from WT mice demonstrated demyelination in their optic nerve (**Figure 4A**), brain (**Figure 4B**) and spinal cord (**Figure 4C**), while as expected control IL-17A^-/-^ mice similar to **Figure 1** (above) showed no CNS demyelination (**Figures 4D–F**). The results of adoptive transfer of WT T cells into the IL-17A^-/-^ mouse provide further evidence that T cells expressing IL-17A contribute to CNS demyelination in the absence of IL-17A production by non-T cells.

### Role of IL-17R in HSV-IL-2-induced demyelination

**Figure 5:**
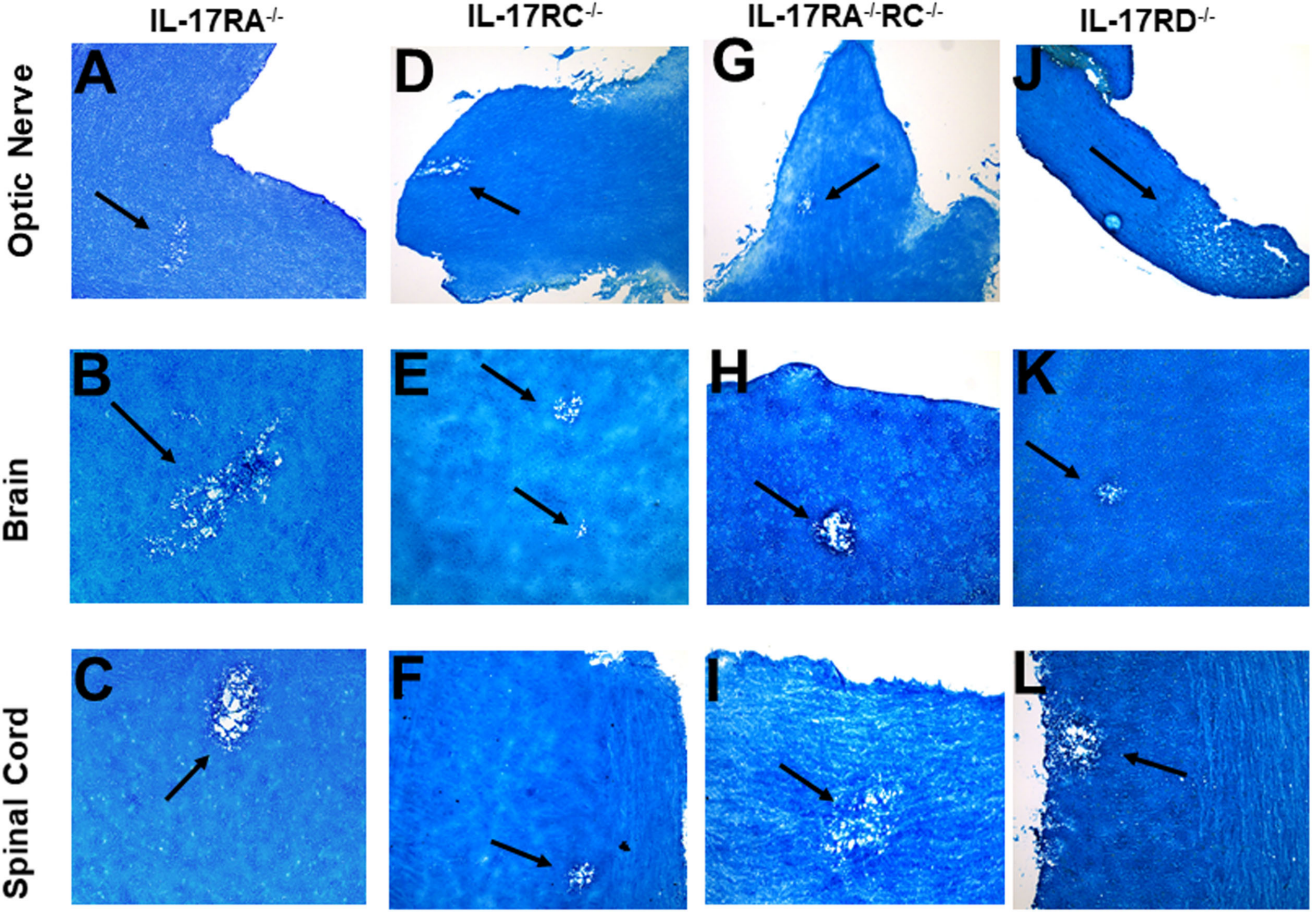
Role of IL-17 receptors in HSV-IL-2 induced CNS demyelination. Five female IL-17RA^-/-^ **(A, B, C)**, IL-17RC^-/-^ **(D, E, F)**, IL-17RA^-/-^RC^-/-^ **(G, H, I)** and IL-17RD^-/-^ **(J, K, L)** mice were infected ocularly with 2 X10^5^ pfu/eye of HSV-IL-2 virus. On day 14 PI, optic nerves **(A, D, G, J)**, brain **(B, E, H, K)**, and spinal cord **(C, F, I, L)** were collected, fixed, sectioned, and stained with LFB. Representative photomicrographs are shown. Arrows indicate areas of demyelination. x10 objective lens was used (100x mag.).

The IL-17 receptor (IL-17R) family has five known members (*i.e.*, IL-17RA, IL-17RB, IL-17RC, IL-17RD, and IL-17RE) (57, 58). However, the functions of these receptors in HSV-IL-2-induced CNS demyelination are not known. IL-17A binds to IL-17RA and IL-17RC (59, 60). IL-17RC is an obligate co-receptor with IL-17RA for signaling induced by IL-17A and IL-17F (61). We first tested if the absence of IL-17RA or IL-17RC blocks CNS demyelination following infection with the HSV-IL-2 virus. Five female IL-17RA^-/-^ and IL-17RC^-/-^ mice in C57BL/6 background were infected with 2 X 10^5^ PFU/eye of HSV-IL-2 virus. On day 14 PI, optic nerves, brain, and spinal cord were isolated and stained with LFB as described in materials and methods. Representative photomicrographs of each type of section are shown in **Figure 5**. Upon ocular infection of IL-17RA^-/-^ and IL-17RC^-/-^ mice with HSV-IL-2, demyelination was detected in all samples of both IL-17RA^-/-^ (**Figures 5A–C**) and IL-17RC^-/-^ (**Figures 5D–F**) infected mice.

Since IL-17A binds to both IL-17RA and IL-17RC, detection of demyelination in IL-17RA^-/-^ and IL-17RC^-/-^ mice may suggest that the other receptor compensates for the missing receptor for binding to IL-17A. Thus, to determine if the absence of both RA and RC molecules is required to block CNS demyelination in infected mice, we generated mice lacking a combination of both IL-17RA and IL-17RC as we described previously (41). We infected IL-17RA^-/-^RC^-/-^ mice with HSV-IL-2 and looked for demyelination in optic nerve, brain, and spinal cord sections of infected mice as above. Similar to only IL-17RA^-/-^ or IL-17RC^-/-^ infected mice, the absence of both RA and RC in IL-17RA^-/-^RC^-/-^ mice did not block demyelination in optic nerves (**Figure 5G**), brain (**Figure 5H**) or spinal cord (**Figure 5I**) of infected mice. Thus, these results suggest that presence of IL-17RA and IL-17RC are not required for induction of CNS demyelination by IL-17A. Finally, we looked at the possible involvement of IL-17RD in CNS demyelination. IL-17RD^-/-^ mice were infected ocularly as described above with HSV-IL-2 virus and optic nerve, brain, and spinal cord of infected mice were isolated on day 14 PI. Isolated tissues were dissected, post-fixed and stained with LFB as above and representative photomicrographs of optic nerve, brain and spinal cord sections from mice infected with HSV-IL-2 virus are shown in **Figure 5 J–L**. Similar to the results stated above we detected demyelination in the optic nerves (**Figure 5J**), brain (**Figure 5K**) and spinal cord (**Figure 5L**) of IL-17RD^-/-^ mice. Collectively, this data suggests that IL-17A plays a critical role in the HSV-IL-2-induced demyelination and that the absence of all the five IL-17 receptors may be necessary to achieve similar blocking of demyelination as with IL-17A^-/-^ infected mice. However, we cannot rule out the possibility that IL-17RB or IL-17RE alone contributes to CNS demyelination or unknown IL-17A receptor contributing to demyelination.

### Role of IL-6, IL-10, and TGFβ in HSV-IL-2 induced demyelination in infected mice

T_H_17 cell development is driven by numerous cytokines including TGF-β, IL-6, and IL-10 (60, 62), and the generation of pathogenic T_H_17 is dependent on the absence of TGFβ, IL-6 and IL-10 (63, 64). Previously, it was shown that levels of TGF-β or IL-6 control the balance between T_H_17 and Treg cells, and their imbalance in favor of the former will break the immune homeostasis in the host and result in the development of autoimmune diseases (65). It was shown that TGF-β secreted by Treg cells was not required for T_H_17 cell development; rather the regulation of IL-2 by Treg cells was found to play an important role in the generation of T_H_17 cells (28). Thus, to test the roles of IL-6, IL-10 and TGF-β in demyelination, IL-6^-/-^, IL-10^-/-^, CD11cdnTGF-RII, and CD4dnTGF-RII female transgenic mice were ocularly infected with HSV-IL-2 as above. The latter two mice express a dominant-negative form of TGF-β RII under the control of either the CD11c (38) or CD4 promoter (39).

**Figure 6:**
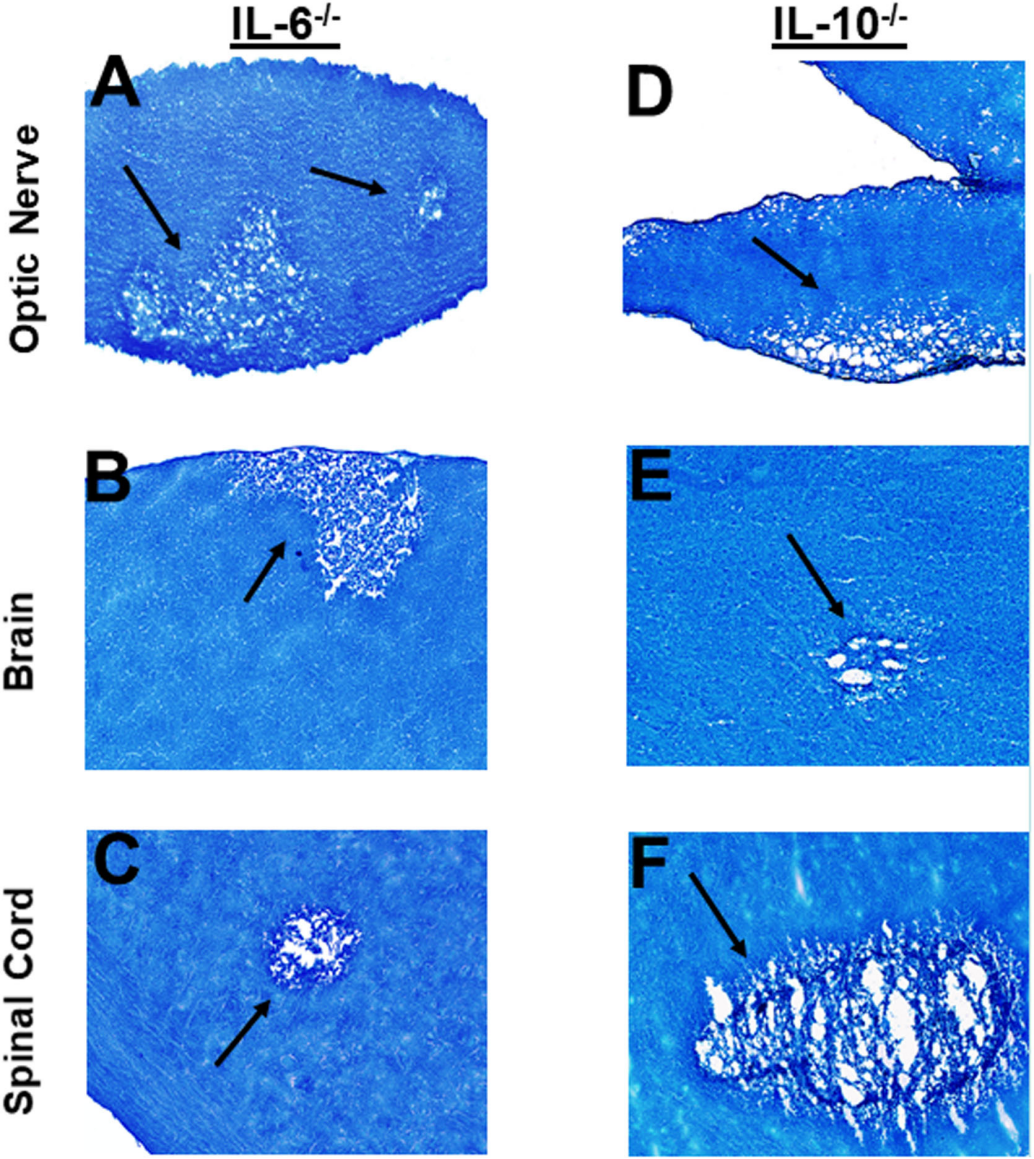
Role of IL-6 and IL-10 in HSV-IL-2 induced CNS demyelination. Five female IL-6^-/-^ **(A, B, C)** and IL-10^-/-^ **(D, E, F)** mice were infected ocularly with 2 X10^5^ pfu/eye of HSV-IL-2 virus. On day 14 PI, optic nerves **(A, D)**, brain **(B, E)**, and spinal cord **(C, F)** were collected, fixed, sectioned, and stained with LFB. Representative photomicrographs are shown. Arrows indicate areas of demyelination. x10 objective lens was used (100x mag.).

Optic nerve, brain, and spinal cord of infected mice that were either IL-6^-/-^ or IL-10^-/-^ were isolated on day 14 PI, sectioned, fixed and stained with LFB as above, and representative photomicrographs of optic nerve, brain and spinal cord sections from infected mice are shown in **Figure 6**. IL-6^-/-^ infected mice showed demyelination in their optic nerves (**Figure 6A**), brain (**Figure 6B**) and spinal cord (**Figure 6C**). Similarly, demyelination was detected in optic nerves (**Figure 6D**), brain (**Figure 6E**) and spinal cord (**Figure 6F**) of IL-10^-/-^ infected mice.

**Figure 7:**
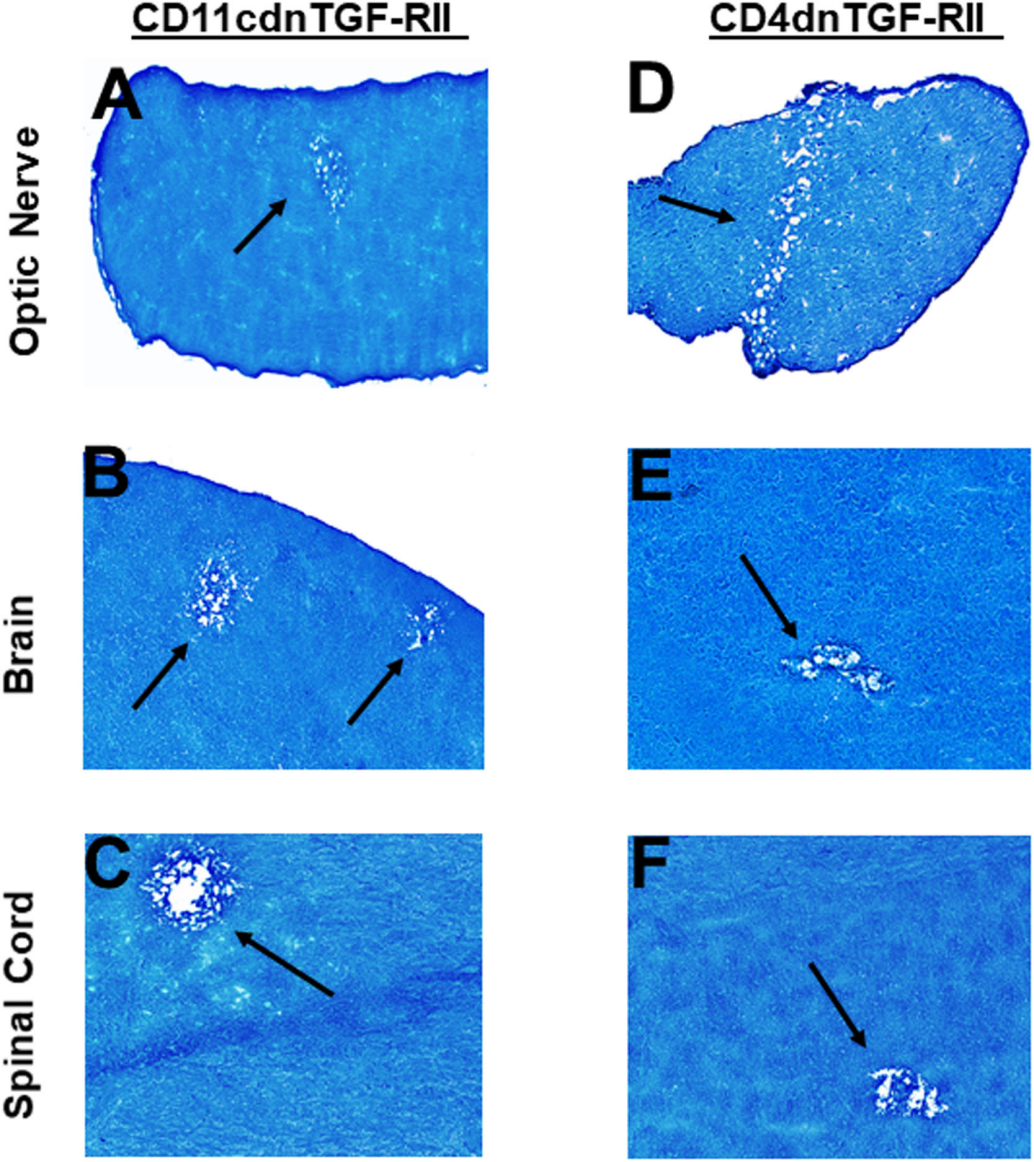
Role of TGFβ in HSV-IL-2 induced CNS demyelination. Five female CD11cdnTGF-RII **(A, B, C)** and CD4dnTGF-RII **(D, E, F)** mice were infected ocularly with 2 X10^5^ pfu/eye of HSV-IL-2 virus. On day 14 PI, optic nerves **(A, D)**, brain **(B, E)**, and spinal cord **(C, F)** were collected, fixed, sectioned, and stained with LFB. Representative photomicrographs are shown. Arrows indicate areas of demyelination. x10 objective lens was used (100x mag.).

Transforming growth factor-beta (TGF-β) is a pleiotropic cytokine that is present on most cell types and acts as a switch to regulate processes such as immune function, cell proliferation and differentiation (66–68). To overcome the developmental problems associated with the generation of TGF-β knockout mice (69–72), we used dominant-negative transgenic mice in which TGF-β signaling by immune cells was blocked (38, 39). In CD11cdnTGF-RII mice, the innate immune cells do not respond to TGF-β ligands, while in CD4dnTGF-RII mice, T-cells do not respond to TGF-β. CD11cdnTGF-RII and CD4dnTGF-RII mice were ocularly infected with HSV-IL-2 virus as above. Optic nerve, brain, and spinal cord of infected CD11cdnTGF-RII and CD4dnTGF-RII mice were isolated on day 14 PI. Isolated tissues were sectioned, fixed, and stained with LFB as above, and representative photomicrographs of optic nerve, brain and spinal cord sections from mice infected with HSV-IL-2 virus are shown in **Figure 7**. CD11cdnTGF-RII infected mice showed demyelination in their optic nerves (**Figure 7A**), brain (**Figure 7B**) and spinal cord (**Figure 7C**). Similarly, demyelination was detected in optic nerves (**Figure 7D**), brain (**Figure 7E**) and spinal cord (**Figure 6F**) of CD4dnTGF-RII infected mice.

These results suggest that the absence of IL-6, IL-10 or TGF-β signaling in the T cells or innate immune cells had no effect in blocking CNS demyelination in HSV-IL-2 infected mice.

### Size and number of plaques in CNS of infected mice

To quantify the extent of the demyelination, we counted the number and measured the size of the observed demyelination plaques in the brains, spinal cords and optic nerves of five females each of WT, IL-17A^-/-^, Rag1^-/-^ + WT T cell, Rag1^-/-^ + IL-17A^-/-^ T cell, IL-17A^-/-^ + WT T cell, IL-17RA^-/-^, CD11cdnTGF-RII, CD4dnTGF-RII, IL-6^-/-^, and IL-10^-/-^ mice described above, and we counted the number and the size of observed plaques in the optic nerve, brain and spinal cord of infected mice as we described previously (24, 73). The data are shown as the number of sections showing demyelination plaques per total stained sections as well as area of demyelination per section (**Figure 3**). As expected, no plaques were detected in optic nerves, brain, and spinal cord of IL-17A^-/-^ infected mice (**Figure 3**).

In contrast to IL-17A^-/-^ mice, Rag1^-/-^ + WT T cell, Rag1^-/-^ + IL-17A^-/-^ T cell, IL-17RA^-/-^, IL-6^-/-^, IL-10^-/-^, CD11cdnTGF-RII, and CD4dnTGF-RII infected mice had similar number of plaques per optic nerve section as WT mice (**Figure 3A**). However, IL-17A^-/-^ mice that received T cell from WT mice had lower number of plaques per section of optic nerve (**Figure 3A**). Similar to lower number of plaques in IL-17A^-/-^ + WT T cell mice, these mice also had lower plaque size than other infected mice (**Figure 3B**, IL-17A^-/-^ + WT T cell group). Plaque size in Rag1^-/-^ + WT T cell, Rag1^-/-^ + IL-17A^-/-^ T cell, IL-17A^-/-^ + WT T cell, IL-17RA^-/-^, IL-6^-/-^, IL-10^-/-^, CD11cdnTGF-RII, and CD4dnTGF-RII mice was similar to that of WT mice (**Figure 3B**, P>0.05). The number of plaques (**Figure 3C**) and size of plaques (**Figure 3D**) in the brain of infected mice were also determined as above. The number of plaques in the brain of all groups of infected mice, including IL-17A^-/-^ mice that received T cells from WT mice, were similar to that of WT mice (**Figure 3C**, P>0.05). Similar to the number of plaques, the severity of plaque size in the brain section of the above infected mice were similar to each other in comparison to WT mice (**Figure 3D**, P>0.05). The number of HSV-IL-2-induced plaques detected in the spinal cord was similar between WT mice and Rag1^-/-^ + WT T cell, Rag1^-/-^ + IL-17A^-/-^ T cell, IL-17RA^-/-^, IL-6^-/-^, IL-10^-/-^, CD11cdnTGF-RII, and CD4dnTGF-RII mice (**Figure 3E** **, P>0.05)**. In contrast, less plaques were detected in the spinal cord of IL-17A^-/-^ mice that received T cells from WT mice than other group of infected mice (**Figure 3E** P<0.05). Finally, no significant differences were observed in the size of plaques detected in the spinal cord of all groups of mice compared with WT control mice except IL-17A^-/-^ mice that received T cells from WT mice (**Figure 3F**, P>0.05).

Overall, except for the lower number of plaques and size of plaques in the optic nerve and spinal cord, but not brain, of IL-17A^-/-^ mice that received T cells from WT mice, the absence of IL-17RA, IL-6, IL-10, and TGFβ did not affect the number or size of plaques in the optic nerve, brain or spinal cord of infected mice compared with WT mice. Thus, this study suggests that both IL-17A-expressing T cells and non-T cells contribute to CNS demyelination.

### Gene expression profiles in brain and spinal cord of HSV-IL-2 infected IL-17A^-/-^ mice after T cell transfer

**Figure 8:**
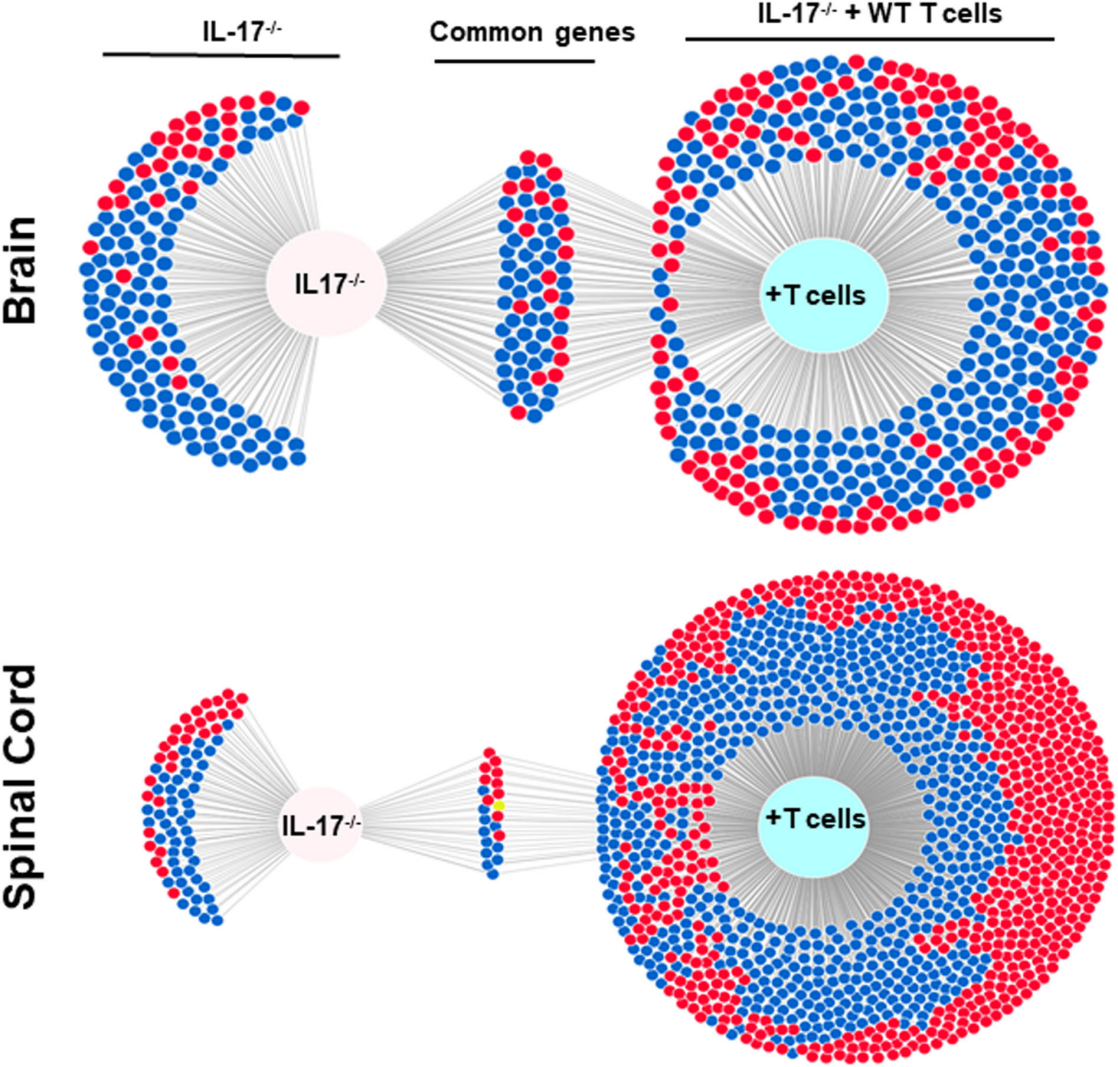
Venn diagram of upregulated, downregulated, and common genes in brain and spinal cord of infected mice. Five IL-17A^-/-^ mice with and without T cell transfer from 5 WT mice were infected with 2 X10^5^ pfu/eye of HSV-IL-2 virus and T cells from the brain and spinal cord of infected mice on 14 days PI were isolated. Brains and spinal cords from 5 naive mice of the same age were also isolated and used to normalize gene expression in infected mice. The transcriptomes of brain and spinal cord of IL-17A^-/-^ infected mice were compared with the corresponding tissue in mice that received T cells from WT mice. Venn diagram shows the numbers of genes uniquely or commonly detected in brain and spinal cord of infected IL-17A^-/-^ mice with and without T cells transfer. Red dots show upregulated genes, while blue dots show downregulated genes. Data are based on three independent replicates.

The above results suggest that IL-17A^-/-^ mice have no demyelination in their CNS, while transfer of isolated T cells from WT mice to recipient IL-17A^-/-^ mice caused CNS demyelination (**Figure 4**). To further characterize changes induced in the IL-17A^-/-^ mice by T cell transfer that leads to CNS demyelination, we used an RNA-seq-based transcriptome analysis approach. T cells from spleens of naive female WT C57BL/6 mice were isolated using magnetic beads and the cells injected intraperitoneally into IL-17A^-/-^ recipient mice. Two weeks after the adoptive transfer, the recipient IL-17A^-/-^ mice as well as control IL-17A^-/-^ mice were infected ocularly with HSV-IL-2 virus. Fourteen days after infection, the mice were sacrificed and total cells from brains and spinal cords of infected mice were isolated. Total RNA from isolated cells from brain and spinal cord of infected mice was extracted and libraries were prepared and sequenced as we described in Materials and Methods. As shown in **Figure 8** (brain), the transcriptome analysis of the brain of infected IL-17A^-/-^ mice showed an upregulation of expression levels of 29 genes, (greater than 2-fold increase; in red), while the brain of T cell recipient infected IL-17A^-/-^ mice, 149 genes were upregulated. Downregulation of 109 genes was found in IL-17A^-/-^ brain (greater than 2-fold decrease, p<0.05; in blue) and 271 genes in brain of recipient IL-17A^-/-^ mice. There were 20 and 50 common genes between the two groups that were upregulated (in red) and downregulated (in blue), respectively. For the spinal cord (**Figure 8**) of infected IL-17A^-/-^ mice, 30 genes were upregulated (in red), and 49 genes were downregulated (in blue), while in the T cell transfer group, 602 genes were upregulated (in red) and 616 downregulated (in blue). There were 10 and 11 common genes between the two groups that were upregulated and downregulated, respectively.

**Figure 9:**
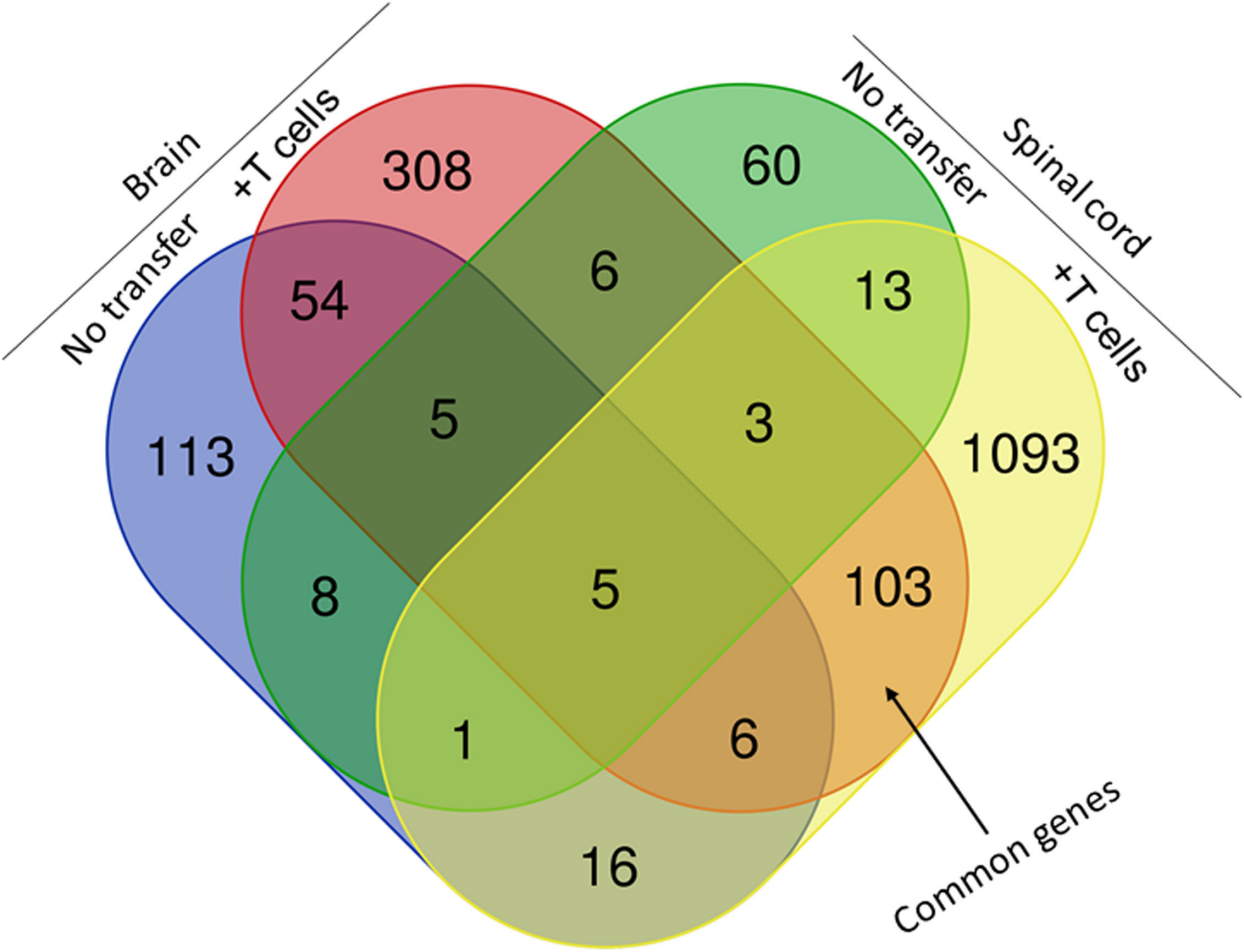
Comparison of transcriptome analysis of upregulated and downregulated common genes in cells isolated from brain and spinal cord of infected mice with demyelination. The transcriptomes of cells isolated from brains and spinal cords of HSV-IL-2-infected IL-17A^-/-^ mice with and without T cells transfer and described above in **Figure 8** were compared after normalization with that of their uninfected counterparts. In mice that received T cells and showed demyelination, we detected 103 genes in common between the brain and spinal cord. In mice with CNS demyelination 60 of the common genes were upregulated, while 43 genes were down regulated. Data are based on three independent replicates.

**Figure 10:**
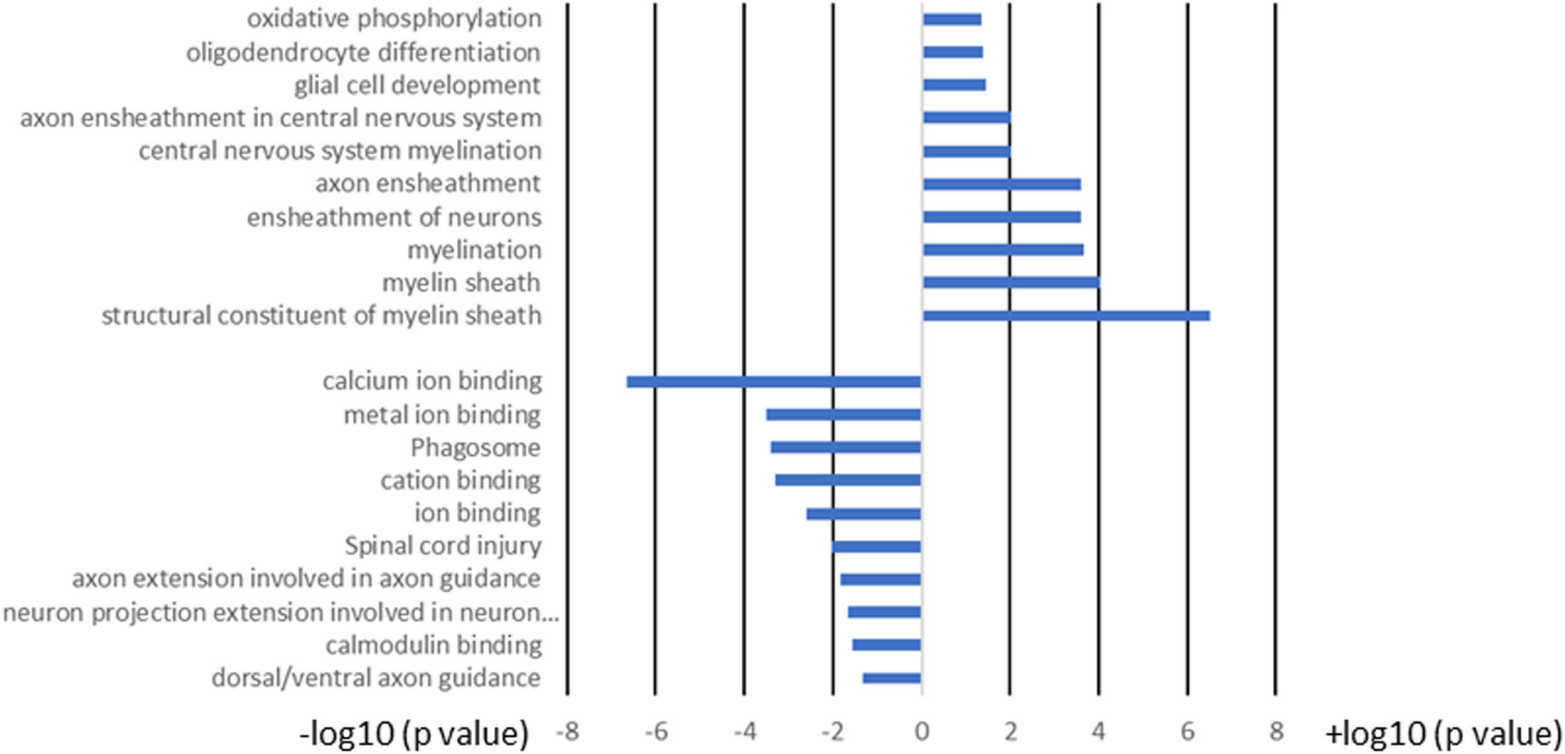
GO term analysis of the pathways for the 103 common genes in brain and spinal cord of T cell recipient mice with demyelination. Bars represent log-converted p-values and minus log-converted p-values for upregulated and downregulated genes, respectively, showing statistical significance for 60 genes that were upregulated and 43 genes that were downregulated in T cell recipient mice with CNS demyelination. The GO terms were ranked according to statistical significance and the top GO terms for the 60 upregulated genes and the top GO terms for the 43 downregulated genes pathways are shown. Data are based on three independent replicates.

We next compared the gene expression patterns between brains and spinal cords of the two groups of infected mice with and without T cell transfer, and a Venn diagram of significantly different genes shared by the four groups and analyzed by Bioinformatics & Evolutionary Genomics is shown in **Figure 9**. There was a total of 103 genes in common between brain and spinal cord of T cells recipient mice (**Figure 9**). From the 103 common genes, 60 genes were upregulated between the two groups, while 43 genes were downregulated. Examination of the gene ontology (GO) terms enriched among the upregulated and downregulated 103 genes in brain and spinal cord of mice that received T cells and had demyelination was carried out using GO profiler (**Figure 10**). In demyelinated brain and spinal cord of infected mice, the GO terms enriched amongst the 60 upregulated genes included (**Figure 10**): “oxidative phosphorylation”; “Oligodendrocyte differentiation”; “glial cell development”; “axon ensheathment in central nervous system”; “central nervous system myelination”; “axon ensheathment”; “ensheathment of neurons”; “myelination”; “myelin sheath”; and “structural constituent of myelin sheath”. The following genes were associated with these pathways: *MOBP, MBP, PLP1, RPS5, MAL, NDUFA7, ARPC1B, GJC3,TSPAN2*, and *ATP5H. PLP1, MBP, MOBP, MAL, GJC3, TSPAN2* are known to be expressed in myelin, and some of gene products are abundant enough to be detected in a proteomic analysis of the mouse myelin (74), which agrees with GO terms related to myelin and myelinating process. Function of the above upregulated genes in brains and spinal cords of demyelinated mice are presented in **Table 1**. Also, proteins encoded by human orthologs of *PLP1, MBP*, and *MOBP* are known to be antigens recognized by CD4^+^ T cells in multiple sclerosis patients (75). Upregulation of *PLP1, MBP, MOBP, MAL, GJC3, TSPAN2* may suggest that some remyelination is occurring in infected mice. This is in line with our previous study that we have shown by electron microscopy (EM) that some remyelination was occurring in brains and spinal cords of HSV-IL-2 infected mice (20). However, no correlation was found with regards to upregulation of *RPS5*, *NDUFA7, ARPC1B*, and *ATP5H* with EAE, MS or other models of CNS demyelination-remyelination.

**Table 1:** Genes associated with upregulation of signaling pathways and their functions in brain and spinal cord of demyelinated mice.

Gene	Gene Function
MOBP (myelin oligodendrocytes basic protein)	Major component of central nervous system
MBP (myelin basic protein)	A major constituent of the myelin sheath
PLP1 (proteolipid protein 1)	A structural constituent of myelin sheath
RPS5 (ribosomal protein S5)	A structural constituent of ribosome
MAL (myelin and lymphocyte protein, T cell differentiation protein)	A highly hydrophobic integral membrane protein, which play a role in the formation, stabilization, and maintenance of glycosphingolipid-enriched membrane microdomains
NDUFA7 (NADH:ubiquinone oxidoreductase subunit A7)	a subunit of the NADH-ubiquinone oxidoreductase (complex I) enzyme, which is the first enzyme complex in the mitochondrial electron transport chain
ARPC1B (actin related protein 2/3 complex, subunit 1β)	A structural constituent of actin cytoskeleton, which mediates actin nucleation.
GJC3 (gap junction protein, γ3)	Forming gap junctions to connect the cytoplasm of two contacting cells
TSPAN2 (tetraspanin 2)	A member of tetraspanin family of membrane protein with 4 transmembrane domains. Tetraspanins regulate the trafficking and function of other membrane proteins
ATP5H (ATP synthase, H^+^ transporting, mitochondrial F0 complex, subunit D)	Involved in mitochondrial ATP synthesis coupled proton transport

The following pathways were associated with the downregulation of the 43 genes in demyelinated brain and spinal cord of infected mice (**Figure 10**): “calcium ion binding”; “metal ion binding”; “Phagosome”; “cation binding”; “ion binding”; “Spinal cord injury”; “axon extension involved in axon guidance”; “neuron projection extension involved in neuron projection guidance”; “Calmodulin binding”; and “dorsal/ventral axon guidance”. The following genes were associated with these downregulated pathways: *AK5, ATP8A2, ATP2B4, C3, CPNE6, DYNC1H1, GM2004, NOS1, NRP2, H2-Q6, H2-Q7, HSPA2, HYOU1, ITGB2, LRP1, PLXNA2, RPS6KA2, SLIT1, SLIT2, TIMP2, XDH, UBR4*, and 11 protocadherin genes, including *PCDH1, PCDH8, PCDHGA2, PCDHGA3, PCDHGA4, PCDHGA5, PCDHGA6, PCDHGA7, PCDHGA9, PCDHGB5*, and *PCDHB9.* Among these genes, *Nrp2, Slit1, Slit2, Plxna2*, and 11 protocadherin genes are involved in neural circuit formation (76–78). Upregulation of *C3, Nrp2, Lrp1* was observed in demyelinating disease (79–81). The products of *C3* and *Lrp1* were also shown to play a role in pathogenesis of multiple sclerosis or EAE (82, 83) and the product of *Nrp2* is involved in the remyelinating process in demyelinating lesions (80). Additionally, *Timp2* and *Nos1* are assumed to be involved in the pathogenesis of multiple sclerosis (84, 85). Function of the above downregulated genes in brains and spinal cords of demyelinated mice are presented in **Table 2**.

**Table 2:** Genes associated with downregulation of signaling pathways and their functions in brain and spinal cord of demyelinated mice.

Gene	Gene function
AK5 (adenylate kinase 5)	A nucleoside monophosphate kinase which catalyzes the reversible phosphorylation between nucleoside triphosphates and monophosphates
ATP8A2 (ATPase, aminophospholipid transporter-like, class I, type 8A, member 2)	A transmembrane protein that have aminophospholipid flippase activity driven by ATP
ATP2B4 (Ca^++^ transporting, plasma membrane 4)	A plasma membrane Ca^++^ pup driven by ATP
C3 (complement component 3)	A complement protein which plays a central role in the classical, alternative and lectin activation pathways of the complement system
CPNE6 (copine VI)	A calcium-dependent membrane-binding protein.
DYNC1H1 (dynein cytoplasmic 1 heavy chain 1)	A subunit of the cytoplasmic dynein complex, which mediate various types of intracellular motility
GM2004 (predicted gene 2004)	A zinc finger protein
NOS1 (nitric oxide synthase 1, neuronal)	A nitric-oxide synthase
NRP2 (neuropilin 2)	A receptor protein that binds to semaphorins, which plays various roles, including neural circuit formation
H2-Q6 and H2-Q7 (histocompatibility 2, Q region locuses 6&7)	A part of MHC class I protein complex
HSPA2 (heat shock protein 2)	Protein folding chaperone
HYOU1 (hypoxia up-regulated 1)	Negative regulation of endoplasmic reticulum stress-induced neuron intrinsic apoptotic signaling pathway
ITGB2 (integrin β2)	Contributes to laminin binding activity
LRP1 (low density lipoprotein receptor-related protein 1)	A transmembrane glycoprotein, which interacts with broad range of secreted proteins and cell surface molecule.
PLXNA2 (plexin A2)	A receptor for semaphorins and involves in axonal guidance
RPS6KA2 (ribosomal protein S6 kinase, polypeptide 2)	A serine/threonine protein kinase, which intermediates the cellular response to several growth factors
SLIT1 and SLIT2 (Slit guidance ligands 1&2)	A cell surface receptor for Robo family receptors, which plays roles in axon guidance
TIMP2 (tissue inhibitor of metalloproteinase 2)	A metalloendopeptidase inhibitor
XDH (xanthine dehydrogenase)	An enzyme that catalyzes xanthine dehydrogenation and oxidation
UBR4 (ubiquitin protein ligase E3 component n-recognin 4)	Ubiquitin protein ligase
PCDH (protocadherin) family (i.e., PCDH1, PCDH8, PCDHγA2, PCDHγA3, PCDHγA4, PCDHγA5, PCDHγA6, PCDHγA7, PCDHγA9, PCDHGβ5, PCDHβ9	Involved in cell adhesion

Overall, amongst the 103 genes in common between the brain and spinal cord of T cells recipient mice with demyelination (**Figures 9**, **10**), two pathways were identified that may have played a detrimental and regenerative role in CNS demyelination based on the literature. One is “neuron projection extension involved in neuron projection guidance” which was downregulated in T cell transferred groups, and another pathway is “ensheathment of neurons” which is upregulated in T cell transferred groups.

## Discussion

Multiple sclerosis (MS) is a neurodegenerative disease that affects approximately 400,000 individuals in the United States with symptoms ranging from relatively benign to overwhelming (86, 87). While the cause of MS remains elusive, it is generally thought that MS is an autoimmune disease, perhaps initiated by a viral infection, that attacks and degrades the myelin sheath (88). Numerous viruses have been proposed as causative agents. Various herpes viruses have been implicated as the trigger for an autoimmune response leading to MS, including HSV-1, HSV-2, HCMV, EBV, HHV-6, and HHV-7 (89–91), although other studies dispute these findings (92, 93). Many non-herpes viruses also have been implicated in MS. At present, the most widely used animal model for the study of MS is EAE. EAE is induced, usually in mice and rats, by immunization with adjuvant plus myelin or myelin components (94–97). In addition to EAE, JC virus (JCV), Semiliki forest virus (SFV), human T lymphotropic virus (HTLV-1), Theiler’s murine encephalomyelitis virus (TMEV), and mouse hepatitis virus (MHV) have all been used as experimental animal models of CNS demyelination (98–104). The above animal models of MS are based on using either the viral model (105) or the direct autoimmune model (106) to initiate disease. The model of MS that we have developed in our Lab to study CNS demyelination incorporates both a viral and an immune component (20, 21, 24, 43, 73, 107, 108). In our model, neither HSV-1 nor IL-2 alone causes demyelination in mice. However, when we delivered IL-2 using the viral vector (HSV-IL-2), an MS-like pathology was observed in infected mice. Previously, we compared the HSV-IL-2 model of CNS demyelination with the MOG_35–55_, MBP_35–47_, and PLP_190–209_ models of EAE using standardized protocols and female C57BL/6 mice of the same age (73). In this study we have shown that mice with HSV-IL-2-induced and MOG-induced demyelinating diseases demonstrated a similar pattern and distribution of demyelination in their brain, spinal cord, and optic nerves, while no demyelination was detected in the optic nerves of MBP- and PLP-injected mice.

T cells are involved in CNS demyelination (20, 24) and T_H_17 cells have also been shown to play a major role in many of the autoimmune diseases (48–56, 109). Thus, in this study we looked at the role of IL-17A in an HSV-IL-2 model of CNS demyelination. IL-17A has been implicated in the pathogenesis of many common autoimmune disorders, including MS, as well as rheumatoid arthritis (RA), psoriasis, and inflammatory bowel disease (48–56). Similar to previous studies (48–56), we have shown now that mice lacking the IL-17A gene are resistant to CNS pathology. The central importance of IL-17A in inflammatory conditions is illustrated by the recent approval of two monoclonal antibody therapies that inhibit interleukin-17A for treatment of psoriasis, spondyloarthropathies, psoriatic arthritis and ankylosing spondylitis (110), although both these antibody therapies have significant side effects. IL-17A is produced by many cell types including T cells, ILCs, CD4^−^CD8^−^ T cells, γδT cells, invariant natural killer (iNK) T cells, NK cells, neutrophils, mast cells and B cells (26, 27, 29–34). In this study we transferred T cells from IL-17A^-/-^ mice to Rag^-/-^ mice as well as T cells from WT mice to IL-17A^-/-^ mice and showed both T cells and non-T cells are contributing to CNS demyelination. Among the non-T cells, we recently reported a primary role for ILC2 cells but not ILC1 or ILC3 in our model of CNS demyelination (111). In this study we have shown that the absence of ILC2 blocked CNS demyelination in infected mice. Thus, our current study and our previous study (111) suggests that T cells and ILC2 producing IL-17A are contributing to CNS demyelination.

The IL-17 family of cytokines in both mice and human contains five members of the IL-17 receptor (IL-17R) family—IL-17RA, IL-17RB, IL-17RC, IL-17RD, and IL-17RE (57, 58). IL-17A and IL-17F both bind to IL-17 receptors A (IL-17RA) and C (IL-17RC), whose engagement activates mitogen-activated protein kinases (MAPKs), nuclear factor-kappa B (NF-κB), and CCAAT-enhancer-binding protein (C/EBP) signaling pathways through the adaptor proteins Act1 and TRAF6 (59, 60). Thus, IL-17RC is an obligate co-receptor with IL-17RA for signaling induced by IL-17A and IL-17F. In addition, IL-17RC is required for IL-17A- and IL-17F-dependent signaling and has been implicated in the pathogenesis of EAE (61). In this study we looked at the role of IL-17RA, IL-17RC, and IL-17RD in CNS demyelination using a knockout for each gene. HSV-IL-2 infected IL-17RA^-/-^, IL-17RC^-/-^, or IL-17RD^-/-^ mice all showed CNS demyelination, suggesting that other member of IL-17R compensate for the absence of the specific receptor. We also generated mice lacking both IL-17RA and IL-17RC, and the absence of both RA and RC did not block CNS demyelination. The severity of demyelination between different knockout mice lacking one or two of the receptor genes was not affected compared with WT infected mice. However, the functions of IL-17 receptors are not well characterized, and our results may suggest that the absence of all five receptors may be required to block CNS demyelination. Study is in progress to make a knockout mouse lacking all five receptor genes.

RNA-seq analysis further defined the role of IL-17A response against HSV-IL-2 induced demyelination. In our study we have shown the absence of CNS demyelination in IL-17A^-/-^ mice following ocular infection with HSV-IL-2 virus, while transfer of T cells from WT mice to recipient IL-17A^-/-^ mice caused CNS demyelination in infected mice. The transcriptome analysis in brains and spinal cords of infected IL-17A^-/-^ mice with and without T cells transfer were compared after normalization with that of uninfected mice T cells. In both the brains and spinal cord of IL-17A^-/-^ mice that received T cells transfer and demonstrated demyelination, 60 genes were upregulated, while 43 genes were downregulated. Based on the literature these genes are involved in both demyelination and remyelination. GO term analysis identified the two major pathways of “neuron projection extension involved in neuron projection guidance” which was downregulated in the T cell transferred group and “ensheathment of neurons” which is upregulated in T cell transferred group. These pathways are known to be involved in CNS demyelination in both MS and EAE models of MS (76–85).

Our results have shown the importance of IL-17A in HSV-IL-2-induced CNS demyelination. Various molecules including TGF-β, IL-6, and IL-10 are contributing to the development of T_H_17 cells (60, 62). At the same time, the absence of these three cytokines can result in the generation of pathogenic T_H_17 cells (63, 64). In this current study, the lack of IL-6 or IL-10 and the absence of TGF-β expression in DCs or T cells did not alter the level of demyelination in the CNS of infected mice. Overall, our results suggest that IL-6, IL-10 or TGF-β are not required for T_H_17 cell induced demyelination and expression of IL-2 by HSV-IL-2 plays an important role in the generation of pathogenic T_H_17 cells as was reported previously (28).

## Data availability statement

The datasets presented in this study can be found in online repositories. The names of the repository/repositories and accession number(s) can be found below: GSE219020 (GEO).

## Ethics statement

The animal research protocols were approved by the Institutional Animal Care and Use Committee of Cedars-Sinai Medical Center (Protocols # 6134 and 9833).

## Author contributions

SH and HG conceived the studies. SH, SW and HM performed experiments and generated primary data, including developing methodology, validation, and data curation. SH, SW, HM and HG performed formal analysis and visualization. SH, SW, SM-K and RL assisted in animal colony maintenance and performing mouse experiments. SW and SH performed and analyzed transcriptional profiling data. SH, UJ, SM-K and HG contributed to writing the manuscript. All authors contributed to reviewing and editing the final manuscript. HG was responsible for project supervision, administration, and funding acquisition. All authors contributed to the article and approved the submitted version.
